# Exons 1 and 10 Polymorphisms of LHCGR Gene and IVF Success

**Published:** 2020

**Authors:** Sora Yasri, Viroj Wiwanitkit

**Affiliations:** 1- Private Academic Practice, Bangkok, Thailand; 2- Department of Community Medicine, Dr DY PAtil University, Pune, India

## Dear Editor,

We read the publication on “Evaluation of the Prevalence of Exons 1 and 10 Polymorphisms of LHCGR Gene and Its Relationship with IVF Success” with a great interest (1). Javadi-Arjmand et al. concluded that “It has been revealed that two common SNPs (rs4539842 and rs2293275) in the LHCGR gene are associated with the outcome of IVF in Iranian infertile women (1)”. Basically, the polymorphisms of LHCGR gene result in molecular structure change and it can further affect phenotypic expression. Nevertheless, there are other possible genetic polymorphisms that might be associated with outcome of IVF (2). The examples of those polymorphisms are estrogen, progesterone and follicle stimulating hormone receptor polymorphisms (3). It is hard to verify the observed effects of exons 1 and 10 polymorphisms of LHC-GR gene in the present report by Javadi-Arjmand et al. (1). Further studies to assess possible confounding effects of those genetic factors are required.
