# Supplementary material for: Exons 1 and 10 Polymorphisms of LHCGR Gene and IVF Success
Source: J Reprod Infertil. 2020 Jul-Sep;21(3):228. (PMC7362091)
Supplement: Related texts

# Response to letter to editor

**Authors:** Mohsen Ghadami

**Affiliations:** Department of Medical Genetics, Faculty of Medicine, Tehran University of Medical Sciences

## Dear Editor,

We truly appreciate your consideration upon our manuscript: “Evaluation of the Prevalence of Exons 1 and 10 Polymorphisms of LHCGR Gene and Its Relationship with IVF Success”. As you know, IVF is a complex procedure and many genetic and environmental factors influence this procedure. Among genetic factors, polymorphisms in some particular genes such as FSH receptor, estrogen, LH receptor gene, *etc.* are important in infertile women undergoing IVF. We have chosen one of these important polymorphisms in LHCGR gene that has not been examined specifically in other studies. In our conclusion part, we have mentioned that among three polymorphisms that we have studied and examined in LHCGR, two of them can be suggested only as predictive factors in IVF outcome. We also have mentioned in our article that to confirm our results, first of all, a larger number of infertile women should be analyzed and next, we or other investigators need to do combined assessment on LHCGR and FSHR polymorphisms. Also, it is very important to carry out such studies in different populations and examine other genes associated with IVF outcome.

**Yours Sincerely,**
